# No evidence of direct association between GLUT4 and glycogen in human skeletal muscle

**DOI:** 10.14814/phy2.13917

**Published:** 2018-11-22

**Authors:** Robyn M. Murphy, Marcelo Flores‐Opazo, Barnaby P. Frankish, Andrew Garnham, David Stapleton, Mark Hargreaves

**Affiliations:** ^1^ Department of Biochemistry & Genetics and LaTrobe Institute for Molecular Science LaTrobe University Bundoora Australia; ^2^ Department of Physiology The University of Melbourne Melbourne Australia; ^3^ Laboratory of Exercise and Physical Activity Sciences Department of Physiotherapy University Finis Terrae Santiago Chile; ^4^ School of Exercise & Nutrition Sciences Deakin University Burwood Australia

**Keywords:** GLUT4, Glycogen, skeletal muscle

## Abstract

Previous studies have demonstrated that exercise increases whole body and skeletal muscle insulin sensitivity that is linked with increased GLUT4 at the plasma membrane following insulin stimulation and associated with muscle glycogen depletion. To assess the potential direct association between muscle glycogen and GLUT4, seven untrained, male subjects exercised for 60 min at ~75% *V*O_2_ peak, with muscle samples obtained by percutaneous needle biopsy immediately before and after exercise. Exercise reduced muscle glycogen content by ~43%. An ultracentrifugation protocol resulted in a ~2‐3‐fold enriched glycogen fraction from muscle samples for analysis. Total GLUT4 content was unaltered by exercise and we were unable to detect any GLUT4 in glycogen fractions, either with or without amylase treatment. In skinned muscle fiber segments, there was very little, if any, GLUT4 detected in wash solutions, except following exposure to 1% Triton X‐100. Amylase treatment of single fibers did not increase GLUT4 in the wash solution and there were no differences in GLUT4 content between fibers obtained before or after exercise for any of the wash treatments. Our results indicate no direct association between GLUT4 and glycogen in human skeletal muscle, before or after exercise, and suggest that alterations in GLUT4 translocation associated with exercise‐induced muscle glycogen depletion are mediated via other mechanisms.

## Introduction

It is well recognized from studies in rodents and humans that exercise increases insulin‐stimulated glucose uptake in skeletal muscle (Richter et al. 1982, 1989; Cartee and Holloszy 1990; Wojtaszewski et al. 2000). This is associated with muscle glycogen depletion during exercise (Bogardus et al. 1983; Ivy et al. 1985) and enhanced GLUT4 translocation to the plasma membrane with insulin stimulation in the post‐exercise period (Hansen et al. 1998). Both contraction‐ and insulin‐stimulated glucose transport and GLUT4 translocation are influenced by muscle glycogen concentrations (Host et al. 1998; Derave et al. 1999, 2000; Kawanaka et al. 1999, 2000) and this could be mediated via mechanisms that impact upon signaling pathways responsible for these processes and/or direct interaction between muscle glycogen and GLUT4 vesicles. Indeed, it has been suggested that GLUT4 vesicles may directly associate with glycogen (Coderre et al. 1995), although empirical evidence in support of this hypothesis is lacking. In the present study, we tested the hypothesis that there is a direct association between GLUT4 and glycogen in human skeletal muscle by utilizing two different analytical approaches on muscle samples obtained before and after a single bout of glycogen‐lowering exercise.

## Methods

Seven, healthy untrained male subjects (27 ± 2 years; 75 ± 5 kg, *V*O_2_ peak = 41 ± 2 mL·kg^−1^·min^−1^, mean ±SD) agreed to participate in this study after providing their written, informed consent. The study was approved by the Human Research Ethics Committee of The University of Melbourne. Subjects reported to the laboratory in the morning after an overnight fast and having abstained from alcohol, caffeine, and physical activity for at least 24 h. They completed 60 min of cycle ergometer exercise at a power output requiring ~75% of their previously determined peak pulmonary oxygen uptake (*V*O_2_ peak). Muscle samples were obtained from vastus lateralis by percutaneous needle biopsy before and immediately after exercise. A small part of the sample was frozen in liquid N_2_ and stored for later treatment and analysis, while a second portion was used for single fiber dissection.

### Single fibers

In six subjects, single muscle fiber segments (pre: *n* = 90; post: *n* = 117) were isolated by microscopic dissection and mechanically skinned under paraffin oil as described previously (Murphy et al. 2012). Data were obtained for 30 (pre) and 39 (post) fiber sets, with each set containing three fibers. An average of 17 (range 6–42) fibers per subject per time point were analyzed. Skinned fibers were then washed in 10 *μ*L physiological buffer solution (129 mmol/L K^+^, 1 mmol/L free Mg^2+^, 10.3 mmol/L total Mg^2+^, 90 mmol/L HEPES, 50 mmol/L EGTA, 8 mmol/L ATP, 10 mmol/L creatine phosphate, pH 7.0 and osmolality 295 ± 10 mosm·kg^−1^), and in successive solutions containing amylase (70 ng·*μ*L^−1^; Sigma A4268, PMSF‐treated amylase solution) and 1% Triton X‐100 for 10 min to obtain proteins present as diffusible (Diff), glycogen associated (Amy), membrane associated (Trit) or myofibrillar (remaining in fiber, F‐sk), respectively, as described previously (Murphy et al. 2012). About 5 *μ*L of loading buffer (0.125 mol/L Tris HCl, pH 6.8, 4% SDS, 10% glycerol, 4mol/L urea, 10% mercaptoethanol and 0.01% bromophenol blue) were added to the collected fibers and wash solutions which were analyzed side‐by‐side for GLUT4 content by immunoblotting.

### Muscle analyses

Frozen muscle samples were homogenized in HEPES buffer. A portion was collected for whole muscle analyses and a further portion used to obtain enriched glycogen fractions after a series of ultracentrifugation steps, with the pellet and supernatants collected after final step (200,000 g for 1 h) as described previously (Ryu et al. 2009). Our intent in obtaining an enriched glycogen fraction was to maximize our ability to identify any GLUT4 associated with it. Glycogen concentration was assessed using the amyloglucosidase enzymatic method as used previously (Ryu et al. 2009). GLUT4 content was analyzed by SDS‐PAGE and immunoblotting with a specific antibody (anti‐rabbit, 1:1000, PAA1‐1065, Lot LE147295, Thermo Fisher) in whole muscle homogenates, enriched glycogen fractions (with and without amylase treatment) and supernatants. In selected samples glycogenin, a protein known to be associated with glycogen in skeletal muscle (Murphy et al. 2012), was probed with a polyclonal antibody (Ryu et al. 2009).

### Immunoblotting

Immunoblotting of single fibers and wash solutions was performed by separating protein on 4–15% Criterion Stain‐Free gels (Bio‐Rad, Hercules, CA) as previously described (Murphy et al. 2012). Following transfer, nitrocellulose membranes were blocked and probed with GLUT4 antibody while being constantly rocked overnight at 4C and 2 h at room temperature. Following washes and exposure to the secondary antibody (goat anti‐rabbit IgG‐HRP, 1:20000, 31460, Pierce), chemiluminescent images were captured and quantified using densitometry (Chemidoc MP and ImageLab software, Bio‐Rad, Hercules, CA). The proportion of GLUT4 protein in a given pool was expressed as a percentage of the total GLUT4 in all four of the single fiber fractions, that is, %GLUT4 in Trit = GLUT4 density in Trit/sum densities GLUT4 (Diff + Amy + Trit + FSk).

### Statistics

Data obtained before and after exercise and following wash treatments were compared using paired *t*‐test or analysis of variance, as appropriate, with significance at the *P* < 0.05 level. Data are reported as means ± SEM.

## Results

**Figure 1:**
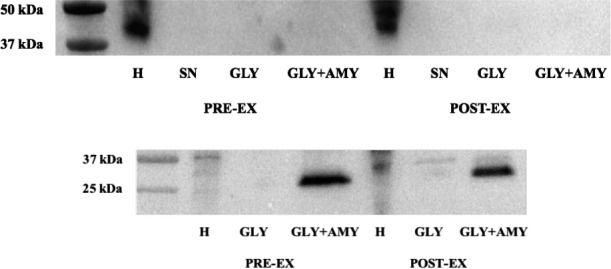
Western blot for GLUT4 (top panel) and glycogenin (bottom panel) on muscle samples collected before (PRE‐EX) and after (POST‐EX) exercise. H – whole homogenate; SN – supernatant from glycogen enrichment protocol; GLY – glycogen‐enriched pellet; GLY+AMY – glycogen‐enriched pellet with amylase treatment.

**Figure 2:**
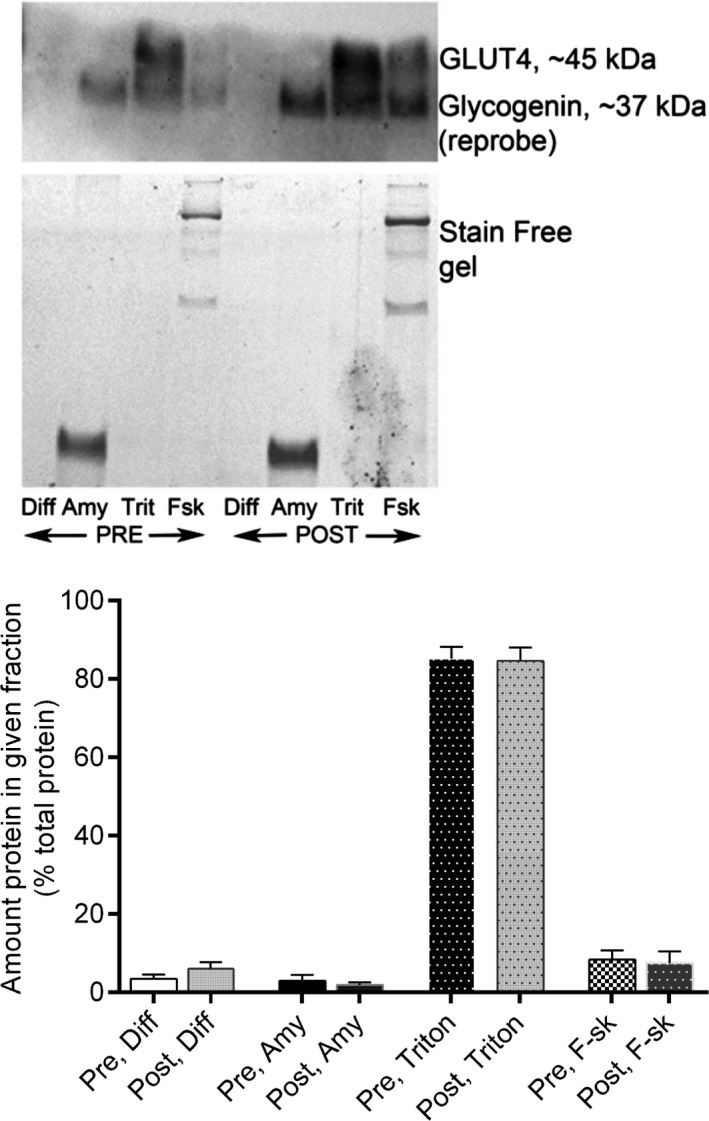
Representative blot of GLUT4 and glycogenin (top panel) and pooled data for GLUT protein content (expressed as % of total GLUT4 pool; bottom panel) in wash solutions (Diff, Amy and Triton) and skinned fibers (F‐sk) before (PRE) and after (POST) exercise.

Exercise reduced muscle glycogen content by ~43% (70.3 ± 4.8 vs. 39.8 ± 5.4 mmol·kg^−1^ wet muscle, *P* < 0.05). The ultracentrifugation protocol resulted in a ~2‐3‐fold enrichment of glycogen concentration. Total GLUT4 content was unaltered by exercise and we were unable to detect any GLUT4 in glycogen fractions, either with or without amylase treatment (Fig. 1). In contrast, we were able to identify glycogenin in the glycogen fraction following amylase treatment (Fig. 1), as has been reported for rat skeletal muscle (Murphy et al. 2012). In skinned muscle fiber segments, there was very little, if any, GLUT4 detected in the wash solutions, except following exposure to 1% Triton X‐100 indicating the membrane–bound nature of the GLUT4 protein (Fig. 2). Amylase treatment of single fibers did not increase GLUT4 in the wash solution and there were no differences between fibers obtained before or after exercise for any of the wash treatments (Fig. 2).

## Discussion

Using two different analytical approaches, our results suggest that there is no direct association between GLUT4‐containing vesicles and glycogen in human skeletal muscle. Recent studies have identified a number of proteins that are associated with skeletal muscle glycogen particles, the so called “glycosome” (Shearer and Graham 2004; Murphy et al. 2012), with roles in glycogen metabolism and potentially wider metabolic regulation. We cannot exclude the possibility of protein‐protein interactions existing between GLUT4‐containing vesicles and glycosomes in vivo that we were unable to detect with our in vitro approaches. A direct link between muscle glycogen content and GLUT4 vesicles has been suggested previously (Coderre et al. 1995), but to date this has not been demonstrated empirically. Thus, our results suggest that other mechanisms operate to mediate the association between muscle glycogen concentration and GLUT4 translocation in response to stimuli such as muscle contraction and insulin (Host et al. 1998; Derave et al. 1999, 2000; Kawanaka et al. 1999, 2000). These could include changes in the activities of key signaling proteins. An obvious candidate is AMP‐activated protein kinase (AMPK), the activity of which has been shown to be increased when muscle glycogen levels are reduced (Wojtaszewski et al. 2003; Steinberg et al. 2006) and which appears to mediate the post contraction/exercise increase in muscle insulin sensitivity (Kjøbsted et al. 2017). It has been suggested that any stimulus (e.g. AICAR, contractions, insulin, hypoxia) that increases muscle glucose uptake results in enhanced sensitivity to subsequent activation, mediated via greater GLUT4 translocation to the cell surface (Geiger et al. 2006). The molecular mechanisms responsible for this phenomenon remain to be fully elucidated. There are also well described links between muscle glycogen and key components of the excitation‐contraction coupling system, notably the sarcoplasmic reticulum, and it is possible that the effects of variations in muscle glycogen concentration are mediated by signaling events secondary to changes in sarcoplasmic calcium levels. In the present study, we obtained muscle samples directly after exercise and the well described increases in muscle insulin sensitivity occur several hours into recovery (Richter et al. 1989; Wojtaszewski et al. 2000), limiting our ability to infer links between these two events. Nevertheless, the key finding of our study remains, that is, that there is not a direct association between GLUT4 and muscle glycogen in human skeletal muscle. Finally, it is worth noting that there have been some studies in which exercise has been shown to increase muscle insulin sensitivity, although muscle glycogen levels were similar between sedentary animals and those that had recovered from exercise (Kawanaka et al. 1999). Thus, there may be some conditions in which there is not a direct causal link between muscle glycogen depletion and insulin action.

In summary, using two different experimental approaches we did not observe a direct association between GLUT4 and glycogen in human skeletal muscle before or after exercise. This suggests that the altered GLUT4 translocation associated with exercise‐induced muscle glycogen depletion is mediated via a mechanism other than a direct glycogen‐GLUT4 interaction.

## Conflict of Interest

None declared.
